# A divided and prioritized experience replay approach for streaming regression^☆^^☆☆^

**DOI:** 10.1016/j.mex.2021.101571

**Published:** 2021-11-12

**Authors:** Mikkel Leite Arnø, John-Morten Godhavn, Ole Morten Aamo

**Affiliations:** a Department of Engineering Cybernetics, Norwegian University of Science and Technology, Trondheim 7491, Norway; b Equinor Research Center, Ranheim 7053, Norway

**Keywords:** Streaming regression, Catastrophic forgetting, Nonstationarity

## Abstract

In the streaming learning setting, an agent is presented with a data stream on which to learn from in an online fashion. A common problem is catastrophic forgetting of old knowledge due to updates to the model. Mitigating catastrophic forgetting has received a lot of attention, and a variety of methods exist to solve this problem. In this paper, we present a divided and prioritized experience replay approach for streaming regression, in which relevant observations are retained in the replay, and extra focus is added to poorly estimated observations through prioritization. Using a real-world dataset, the method is compared to the standard sliding window approach. A statistical power analysis is performed, showing how our approach improves performance on rare, important events at a trade-off in performance for more common observations. Close inspections of the dataset are provided, with emphasis on areas where the standard approach fails. A rephrasing of the problem to a binary classification problem is performed to separate common and rare, important events. These results provide an added perspective regarding the improvement made on rare events.

• *We divide the prediction space in a streaming regression setting*
• *Observations in the experience replay are prioritized for further training by the model’s current error*

## Graphical abstract

**Figure:**
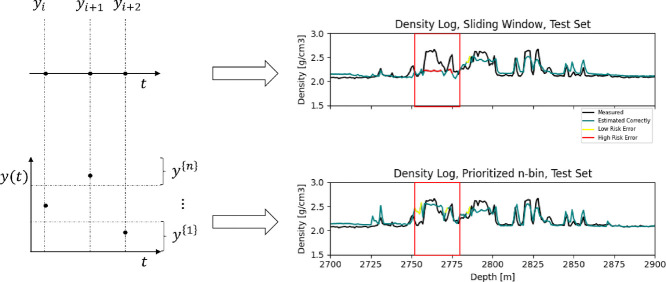

Specification table

**Table:** 

Subject area	*Computer science*
More specific subject area	*Streaming learning*
Method name	*Prioritized n-bin experience replay*
Name and reference of original method	*N/A*
Resource availability	*N/A*

## Introduction

In supervised learning, generalizability is a primary focus during the development of a model. During training, the labels are available, and are used to supervise the learning of a function that maps from inputs to an output. The goal is for the model to learn a function that gives accurate predictions for unseen observations, for which labels are not available. This is the essence of generalization, and relies on the assumption that the unseen observations come from the same distribution as the training observations. However, this assumption fails for many real-word problems. Shifts in independent or dependent variables, an evolving underlying process, and dependence on variables not included in the model are all examples of nonstationarity, which is harmful to the predictive power of such models [1], [2]. Nonstationarity occurs to some extent in most real world data sets, and has been a motivating factor for the paradigm of streaming learning, where a model is presented with a stream of data on which to continuously learn from in an online fashion. This allows adaptation to changing data distribution, although the approach is prone to catastrophic forgetting [3]. In the setting of streaming learning, the goal is to leverage newly available data to adapt to changing environments while still performing well on previous observations [4]. These two objectives might be conflicting, giving rise to the *stability-plasticity* dilemma [5], asking how one can stay stable to irrelevant events, while plastic to new information. This problem has been addressed in several ways, perhaps most commonly by use of experience replay, where new observations from the data stream are mixed with older observations as they become available [6].

Streaming data can occur in many situations, and several problems can be solved by learning from these streams. Classification with dynamic selection of appropriate window [7], and classification using a streaming random forest [8] have been investigated. Clustering of data streams [9], multi-task learning with a global loss function [10], and multiple output linear regression [11] are other examples. The challenges of streaming learning have been discussed in many earlier publications, with emphasis on managing catastrophic forgetting. Reducing the risk of catastrophic forgetting has been approached in several ways, among them regularization, Kirkpatrick et al. [12], Li and Hoiem [13], where weight updates are constrained so previously learned relationships are not erased, and ensembling methods, where multiple models are trained, and their outputs are combined with some form of majority voting [2], [14], [15]. Various experience replay configurations have also been developed for this purpose, among them stream clustering methods to retain valuable information [16], and prioritization of samples to train on [17], where the former has with benefit been applied to streaming classification, and the latter to reinforcement learning. These differ from the aforementioned methods, as they focus on which observations to retain and train on rather than on the model itself.

In the current work, a divided and prioritized experience replay approach for streaming regression is presented to mitigate the effects of catastrophic forgetting while allowing adaptation to nonstationarity. We adopt both the philosophy of retaining relevant knowledge in the replay, along with that of prioritization. A deep neural network (DNN) is trained and validated on historical data. This model serves as a baseline model which is deployed for streaming learning during operation, using this approach. To demonstrate its effect, the method is applied to a real-world dataset, and benchmarked against a standard sliding window approach. The method was first presented in Arnøet al. [18] as a case study. In the current work, however, a thorough comparison to the standard sliding window approach for streaming regression is given, with focus on rare, important events, and discussions of areas where the standard sliding window fails.

The paper is structured as follows: Section 2 presents the methods used in this work, and Section 3 presents our results in a case study, comparing our method with a standard sliding window. Lastly, Section 4 offers conclusions.

## Method

The selected model architecture for this work was a DNN. DNNs consist of multiple layers of interconnected neurons with nonlinear activation functions. This allows extraction of latent, possibly nonlinear features within the data. First, we provide notation for the DNN:

$$\begin{array}{cc} L & :\text{number}\;\text{of}\;\text{layers}\;\text{in}\;\text{the}\;\text{DNN}\\ m & :\text{number}\;\text{of}\;\text{observations}\\ f & :\text{number}\;\text{of}\;\text{features}\\ x & :\text{features}\\ y & :\text{target}\;\text{variable}\\ s_{l} & :\text{number}\;\text{of}\;\text{neurons}\;\text{in}\;\text{layer}\;l\in 1,\ldots ,L\\ \left(x_{i},y_{i}\right) & :i\text{th}\;\text{observation,}\;i\in 1,\ldots ,m\\ w^{\left[l\right]} & :\text{trainable}\;\text{weight}\;\text{matrix}\;\text{for}\;\text{layer}\;l\\ b^{\left[l\right]} & :\text{trainable}\;\text{bias}\;\text{vector}\;\text{for}\;\text{layer}\;l \end{array}$$

and

(1) $$x=\left[\begin{array}{cccc} \vdots & \vdots & & \vdots \\ x_{1} & x_{2} & \ldots & x_{m}\\ \vdots & \vdots & & \vdots \end{array}\right]\;\in R^{f\;\text{x}\;m},$$

(2) $$y=\left[\begin{array}{cccc} y_{1} & y_{2} & \ldots & y_{m} \end{array}\right]\;\in R^{1\;\text{x}\;m},$$

(3) $$w^{\left[l\right]}=\left[\begin{array}{ccc} \ldots & w_{1}^{[l]⊤} & \ldots \\ \ldots & w_{2}^{[l]⊤} & \ldots \\ & \vdots & \\ \ldots & w_{s_{l-1}}^{[l]⊤} & \ldots \end{array}\right]\;\in R^{s_{l-1}\;\text{x}\;s_{l}},$$

(4) $$b^{\left[l\right]}=\left[\begin{array}{c} b_{1}^{\left[l\right]}\\ b_{2}^{\left[l\right]}\\ \vdots \\ b_{s_{l}}^{\left[l\right]} \end{array}\right]\;\in R^{s_{l}\;\text{x}\;1}.$$

To make a prediction on a single observation of inputs $x_{i}$, the input is mapped to ${\hat{y}}_{i}$ by forward propagation through the DNN:

(5) $$z_{i}^{\left[l\right]}=w^{[l]⊤}a_{i}^{\left[l-1\right]}+b^{\left[l\right]}\;l=1,\ldots ,L,$$

(6) $$a_{i}^{\left[l\right]}=g^{\left[l\right]}\left(z_{i}^{\left[l\right]}\right)\;l=1,\ldots ,L-1,$$

(7) $$a_{i}^{\left[l\right]}=\max\left\{\gamma z_{i}^{\left[l\right]},z_{i}^{\left[l\right]}\right\}\;l=1,\ldots ,L-1,$$

(8) $${\hat{y}}_{i}=a_{i}^{\left[L\right]}=z_{i}^{\left[L\right]}.$$

Eq. (5) describes the linear part of the forward propagation, mapping from one layer to the next throughout the DNN. This starts at $a_{i}^{\left[0\right]}=x_{i}$. At each hidden layer, these linear combinations are passed through nonlinear activation functions $g^{\left[l\right]}$, as described in Eq. (6). In this work, the leaky ReLU activation functions given in Eq. (7) are used, where γ is the slope in the left half plane. Lastly, the output layer outputs the prediction, which for this work is a real number, resulting in a regression layer. This is described in Eq. (8).

For learning on a mini-batch of size $m_{b}$, inputs $x_{b}$ and outputs $y_{b}$ are used. The forward propagation for the mini-batch is given by:

(9) $$z^{\left[l\right]}=w^{[l]⊤}a^{\left[l-1\right]}+b^{\left[l\right]}1\;l=1,\ldots ,L,$$

(10) $$a^{\left[l\right]}=g^{\left[l\right]}\left(z^{\left[l\right]}\right)\;l=1,\ldots ,L-1,$$

(11) $$a^{\left[l\right]}=\max\left\{\gamma z^{\left[l\right]},z^{\left[l\right]}\right\}\;l=1,\ldots ,L-1.$$

(12) $${\hat{y}}_{b}=a^{\left[L\right]}=z^{\left[L\right]},$$

where $1\in R^{1\;\text{x}\;m_{b}}$ is a row vector of ones broadcasting the bias term to each observation of the mini-batch.

The weight initialization is He normal [19], which pulls the weights in each layer from a truncated normal distribution with mean μ=0 and standard deviation $\sigma =\sqrt{\frac{1}{s_{\left[l-1\right]}}}$. The weight initialization for layer l is then given by:

(13) $$w^{\left[l\right]}\in R^{s_{l-1}\;\text{x}\;s_{l}}∼N([0,{\left(s_{\left[l-1\right]}\right)}^{-1}]).$$

The biases, $b^{\left[l\right]}$, are initialized as zeros. The optimizer used was Adam optimization [20], which adaptively estimates appropriate momentum for the gradient updates.

The parameters $w^{\left[l\right]}$ and $b^{\left[l\right]}$ are iteratively updated by means of a gradient-based optimizer to minimize some cost function describing a distance between true labels and predictions. This is done by finding the gradients of the cost function J, w.r.t the trainable parameters, $\frac{\partial J}{\partial w}$ and $\frac{\partial J}{\partial b}$. For this work the cost is the mean squared error between the true labels and the predictions, defined by:

(14) $$J=\frac{1}{2m_{b}}{\left|\hat{y}-y\right|}^{2}.$$

For supervised learning, the procedure of iteratively updating the trainable parameters, along with other hyperparameters such as model architecture, is typically done repeatedly in a validation process to assess the model’s ability to generalize to unseen observations. However, the ability to generalize relies on the assumption that the unseen observations come from the same distribution as the training data, which for many real-world datasets is an invalid assumption. The target variable may depend on features not included in the model, shifts may occur in the independent or dependent variables, or the underlying process may evolve. These can all be contributors to poor generalization, and are called nonstationarity. In streaming learning, the goal is to bridge the gap between data distributions by adapting to new available observations. For this to work, labels must be available so that supervision of the updates can be achieved. In addition to adaption to changing distributions, it is of interest to ”remember” older, relevant observations.

To stay stable to older observations while being plastic to new ones, the prioritized n−bin experience replay was developed. This experience replay configuration allows retention of observations spanning the prediction space $y\in [y_{\min},y_{\max}]$ by splitting it into bins. We denote the buffer as $D\in R^{n\;\text{x}\;N}$, where n is the number of bins, and N is the capacity of each bin. When observation ($x_{i}$, $y_{i}$) becomes available from the data stream, it will be placed in a bin after assessment of which bin in the prediction space $y_{i}$ belongs to. Subsequently, the oldest observation in the same bin is discarded. Using this configuration, observations spanning the prediction space are retained, eliminating bias towards the distribution of the latest available observations, which occurs in the standard sliding window. Additionally, the mini-batch sampled from the experience replay for further learning is sampled by prioritization using the softmax function, so that each observation is assigned a probability of being sampled for training by:

(15) $$p_{j}=\frac{e^{{\left(y_{j}-{\hat{y}}_{j}\right)}^{2}}}{\sum_{c=1}^{C}e^{{\left(y_{c}-{\hat{y}}_{c}\right)}^{2}}},\;j=1,\ldots ,C,$$

where C=nN is the total number of observations in the replay. Assigning the probabilities based on the softmax of the model’s mean squared error on the observations results in added focus on observations for which the model performs poorly, as they are more likely to be sampled. By combining the prioritized n-bin with the DNN using the Adam optimizer, we obtain our streaming learning algorithm, given in Algorithm 1.

**Algorithm 1:**
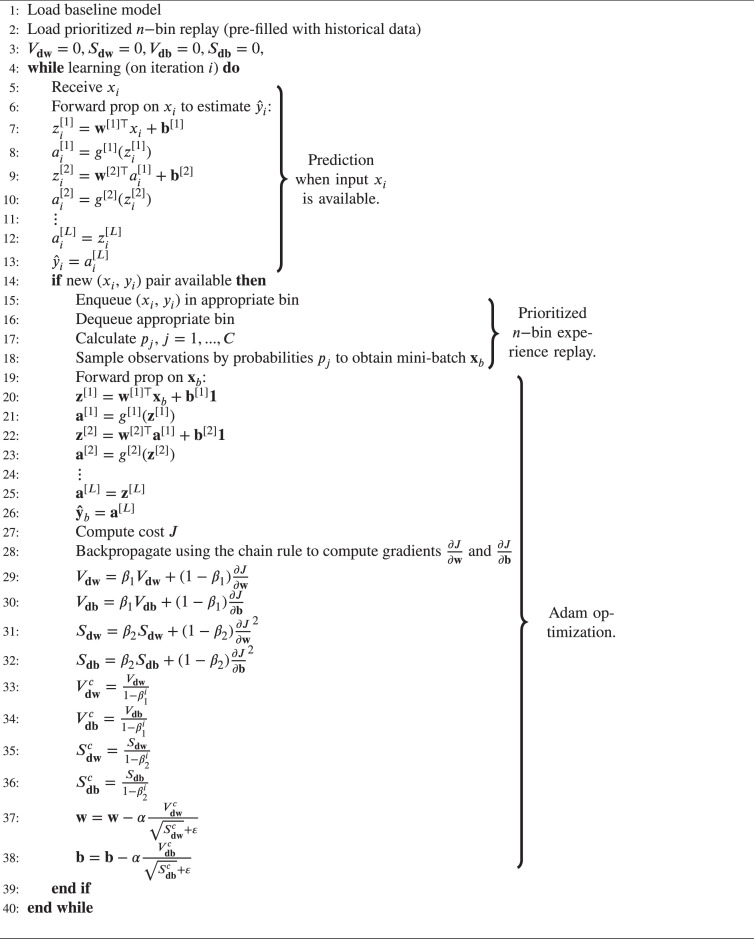
Streaming Learning Using Prioritized n−bin Experience Replay & Adam Optimization

$\beta_{1}$, $\beta_{2}$ and ε are tunable hyperparameters for Adam optimization, $V_{\mathrm{dw}}$ and $V_{\mathrm{db}}$ are biased first moment estimates, and $S_{\mathrm{dw}}$ and $S_{\mathrm{db}}$ are biased second raw moment estimates. Superscript c denotes their bias-corrected counterparts. α is the learning rate.

## Case study

### Problem description

The method presented in this work was developed in order to estimate the density of drilled lithology. This is traditionally measured using the density logging tool, which is a specialized logging while drilling (LWD) tool. Determining the density of the drilled lithology is of interest for several reasons, among them best-practice selection of drilling parameters to ensure a safe and efficient operation. Especially, accurate separation of high-density and low-density lithology can reduce the risk of dysfunctions like buckling, severe doglegs, washout and vibrations, resulting in lost time. However, the density log is delayed due to its placement behind the bit, making mechanical drilling parameters the earliest indicators of change in drilled lithology, although it is very difficult for humans to directly interpret density from these.

To eliminate the density log delay, we propose to estimate a virtual density log using parameters available at the bit, i.e. mechanical drilling parameters. Since the true label of the density log is available after the distance from the log to the bit is drilled, we can pair the delayed density log with drilling parameters measured earlier at the same depth to obtain complete input/output observations that can be used to further supervise updates to our model during operation. This turns the problem into a streaming regression problem with delayed labels. Drilling data from wells on a field operated by Equinor was used for this work. After data cleaning and removal of irrelavant observations, the training set contained approximately 740 000 observations from 5 different wellbores, while the validation set contained 365 000 observations from one wellbore. Lastly, the test set contained 227 000 observations from one wellbore. As per standard convention for deep learning, the data was normalized and scaled so that each feature had zero mean and unit variance.

Several mechanical drilling parameters are available during drilling. v is the drilling velocity (ft/hr), w is the weight on bit (lb), T is the torque (lb-ft), ϕ is the drillstring rotation (rpm). The driller may directly control v, w, and ϕ, while T is dependent on a variety of factors, among them rock properties, w, and ϕ. A metric commonly monitored during drilling is the mechanical specific energy, $U_{ms}$, which quantifies the energy required to remove a unit volume of rock. It is independent of the driller’s actions, is different for different lithologies [21], and is given by:

(16) $$U_{ms}=\frac{w}{a_{b}}+\frac{120\pi \cdot \phi \cdot T}{a_{b}\cdot v},$$

where $a_{b}$ is the bit area (in${}^{2}$). To account for weakening of the rock ahead of the bit due to flow through the nozzles, the hydraulic mechanical specific energy [22], $U_{hms}$, can be defined by:

(17) $$U_{hms}=\frac{w}{a_{b}}+\frac{120\pi \cdot \phi \cdot T}{a_{b}\cdot v}+\frac{1154\eta \cdot \Delta p_{b}\cdot q}{a_{b}\cdot v},$$

where η is the hydraulig energy reduction factor, $\Delta p_{b}$ is the bit pressure drop at the nozzle (psi), and q is the flow rate of drilling fluid (gpm). From the available mechanical parameters, we define the input vector of predictors for the DNN, x, as:

(18) $$x={\left[v,\;\phi ,\;w,\;T,\;U_{ms},\;U_{hms}\right]}^{⊤}.$$

### Pre-training and validation

Pre-training and validation of the baseline model was performed in an informal search. Training was performed using Adam optimization, where the training set was randomly shuffled, and divided into mini-batches of size $m_{b}$. The calculated gradients for each mini-batch are noisy estimates of the true gradients of the entire data set, and this additive noise is useful for avoiding getting stuck in poor local minima or saddle points early in training, and for improving generalization [23]. Neural network architecture and training configuration hyperparameters were iteratively tuned on the training and validation set split. Upon completion of this process, the streaming hyperparameters were tuned iteratively on the validation set. These hyperparameters are related to the streaming learning during operation. The density log limits on this field lies in the range 2.0–2.7 (g/cm${}^{3}$). Tuning of the experience replay parameters resulted in 3 bins with limits at 2.115 (g/cm${}^{3}$) and 2.535 (g/cm${}^{3}$), effectively dividing the prediction space so that one bin retains observations below 2.115 (g/cm${}^{3}$), the second between 2.115 (g/cm${}^{3}$) and 2.535 (g/cm${}^{3}$), and the last retains observations above 2.535 (g/cm${}^{3}$). These parameters can be seen in Table 1, which summarizes the hyperparameters. We can also see that the learning rate is decreased by one order of magnitude for the streaming learning, which was necessary in order to facilitate stability.

**Table 1:** Summary of hyperparameters.

Pre-training hyperparameter	Value
Learning rate	$7.5\times \;10^{-5}$
Hidden layers	3
Neurons in hidden layers	12
Mini-batch size	128
Epochs	25
Optimizer	Adam
**Streaming learning hyperparameter**	**Value**
Learning rate	$7.5\times \;10^{-6}$
Experience replay bins	3
Experience replay bin sizes	256
Experience replay bin limits	[2.115, 2.535]
Mini-batch size	16
Epochs	1
Optimizer	Adam

### Test set results

After pre-training and validation of the baseline model, it was deployed for streaming regression during operation on the test set, where the density logging tool is placed 20 m behind the bit. This was done using both the prioritized n-bin experience replay, and a standard sliding window for comparison. As summarized in Table 1, the prioritized n-bin replay consisted of 3 bins, each containing 256 observations at all times. The standard sliding window used for comparison contained the same amount of observations in total, 768. Although the algorithm is run on data in time domain, the density log (along with other LWD tools) are most interesting in depth domain. For this reason, the presented results are converted to depth domain with equidistant points at 1 m resolution by downsampling. At every integer depth, observations within 0.5 m are averaged. Since detection of the hard stringers is important, we evaluate both approaches in terms of mean absolute error (MAE) separately for low density (<2.35 (g/cm${}^{3}$)) and high density (≥2.35 (g/cm${}^{3}$)) observations.

**Fig. 1:**
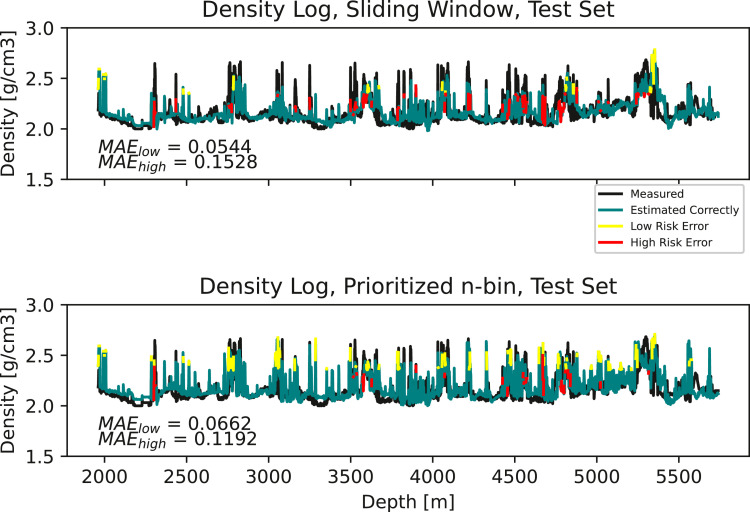
**Top:** Measured and estimated density log using the standard sliding window. **Bottom:** Measured and estimated density log using the prioritized n-bin sliding window.

Fig. 1 illustrates measured and estimated at-bit density vs. depth on the entire test set for both methods. We define a false high density estimate a *low risk* error. If the driller takes action, lowering T and increasing w based on such an estimate, the drilling will simply be sub-optimal. Conversely, we define a false low density estimate as a *high risk* error. If hard stringers are not detected, and action is not taken, risk of dysfunctions such as buckling, severe doglegs, washout and vibrations are increased. On the test set as a whole, the prioritized n-bin experience replay leads to a 22% increase in MAE for true low density observations and a 22% decrease in MAE for true high density observations, compared to the standard sliding window.

We wish to investigate these results in more detail to provide some insight into the effect of applying the prioritized n-replay, both on low density and high density observations. First, we perform paired, two-tailed *t*-tests to investigate the statistical significance of the changes in MAE, through obtaining p-values. The null hypothesis becomes $H_{0}:\mu_{1}=\mu_{2}$, where $\mu_{1}$ is the population mean absolute error for the standard sliding window approach, and $\mu_{2}$ is the population mean absolute error for our method. For this test, we select a significance level of $\alpha_{0}=0.05$. To quantify a standardized *effect size*, we also calculate Cohen’s d. This value, in combination with $\alpha_{0}$, can in turn be used to calculate the statistical power, 1−β, where β is the probability of a type II error. Table 2 summarizes the results of our statistical analysis, where m is number of observations, ${\text{MAE}}_{s}$ is the mean absolute error using a sliding window, ${\text{MAE}}_{n}$ is the mean absolute error using our method, and $\Delta \text{MAE}={\text{MAE}}_{s}-{\text{MAE}}_{n}$. Through the *t*-tests, we confirm the statistical significance at our chosen significance level. Observing Cohen’s d show that for true low density observations, our method leads to a standardized effect size of d=−0.3021 (where the negative sign signifies worsening), while for the true high density observations, d=0.4578. Interpreting these along a continuum, as proposed in literature [24], where 0.2, 0.5, and 0.8 are low, medium and high effect sizes, we see that our approach leads to a small to medium worsening on low density observations, and a medium to large improvement on high density observations.

**Table 2:** Results of power analysis.

True observation value	m	${\text{MAE}}_{s}$ [g/cm${}^{3}$]	${\text{MAE}}_{n}$ [g/cm${}^{3}$]	ΔMAE [g/cm${}^{3}$]	d	p	1−β
<2.35	3422	0.0544	0.0662	−0.01175	−0.3021	$4.63\times \;10^{-35}$	≈1
≥ 2.35	358	0.1528	0.1192	0.03359	0.4578	$2.51\times 10^{-9}$	≈1

**Fig. 2:**
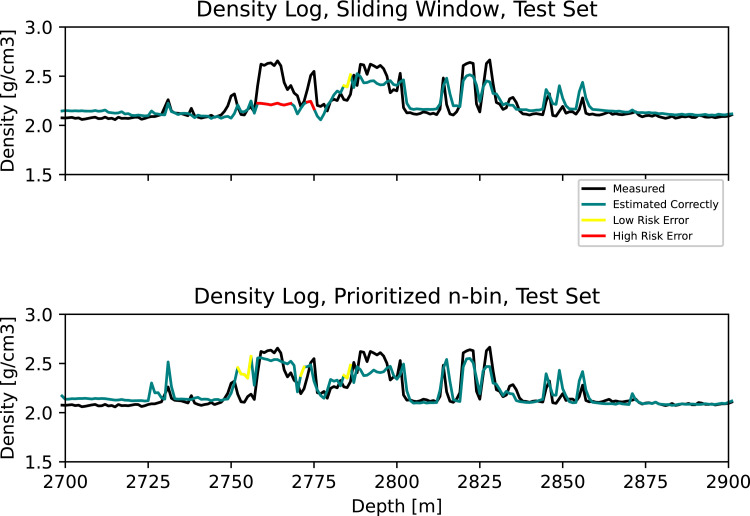
Zoom 1. **Top:** Measured and estimated density log using the standard sliding window. **Bottom:** Measured and estimated density log using the prioritized n-bin sliding window.

**Fig. 3:**
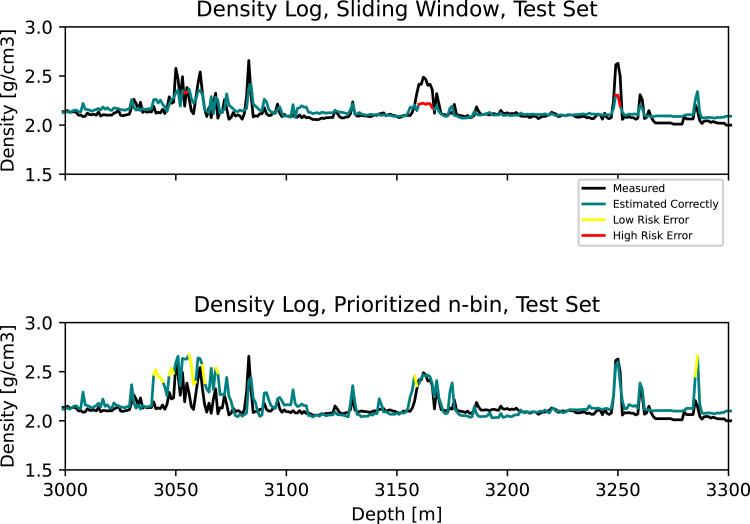
Zoom 2. **Top:** Measured and estimated density log using the standard sliding window. **Bottom:** Measured and estimated density log using the prioritized n-bin sliding window.

**Fig. 4:**
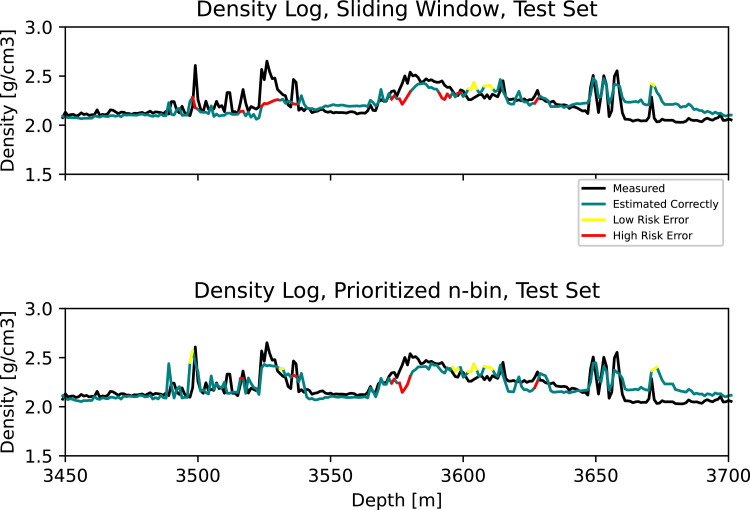
Zoom 3 **Top:** Measured and estimated density log using the standard sliding window. **Bottom:** Measured and estimated density log using the prioritized n-bin sliding window.

**Fig. 5:**
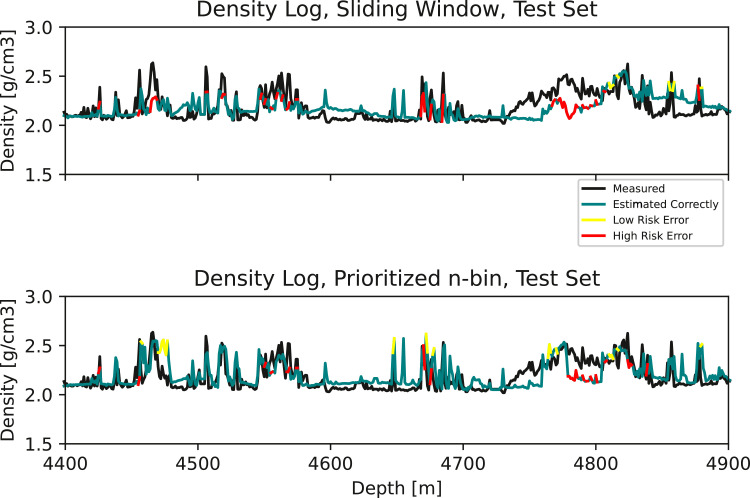
Zoom 4 **Top:** Measured and estimated density log using the standard sliding window. **Bottom:** Measured and estimated density log using the prioritized n-bin sliding window.

Figs. 2–5 are zoomed plots on the test set. In Fig. 2, at 2755 m, a hard stringer occurs. Using the standard sliding window, this event is completely undetected. We can see at 2779 m that the model accurately detects the next stringer. Here, 24 m further down, high density observations from the missed stringer has been passed by the density logging tool (20 m behind), and these observations are available for training in the sliding window. We can assume that the model misses the first stringer since the sliding window only contains observations from the earlier low density observations, resulting in *catastrophic forgetting*. Using our approach, we can see that also the stringer missed by the standard sliding window is detected.

In Fig. 3, we can see that the sliding window misses the stringer at 3157–3170 m, along with the one at 3250 m. Our approach accurately detects these events. At the same time, we observe that some observations at approximately 3050 m and 3285 are overestimated. In Fig. 4, stringers at 3500 m and 3523–3536 m are missed by the sliding window, but detected by our approach. Lastly, Fig. 5 shows missed stringers by the sliding window at 4450–4530 m, along with a poor transition to low density observations at approximately 4850 m. These are all improved using our approach. However, we observe some false high estimates at 4650–4700 m.

**Fig. 6:**
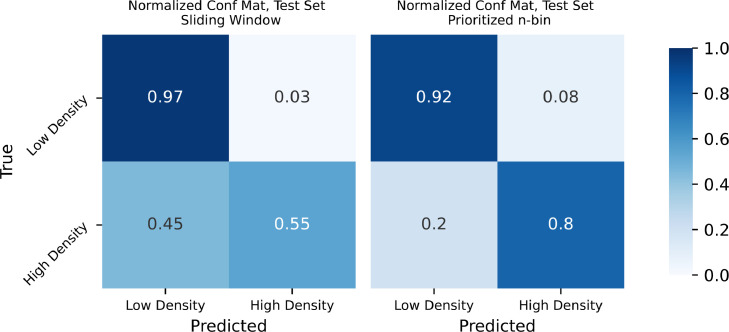
Confusion matrices. **Left:** Standard sliding window. **Right:** Prioritized n-bin method.

**Fig. 7:**
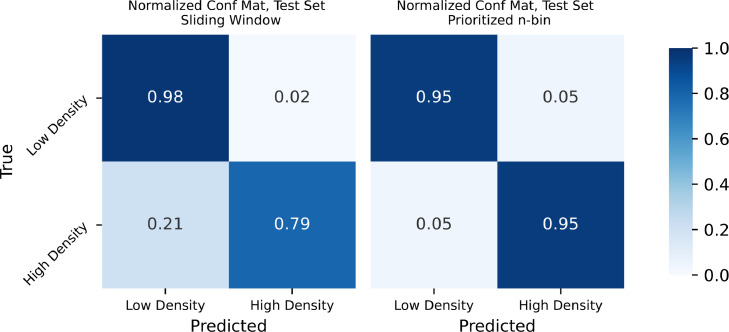
Confusion matrices applying a 1 m acceptance. **Left:** Standard sliding window. **Right:** Prioritized n-bin method.

Since the density log is naturally divided into low- and high density observations, we rephrase the problem into a binary classification problem. Although this is an oversimplification, it adds to the previous analysis in terms of detection of hard stringers, and high risk/low risk errors, as defined previously. As can be seen from m in Table 2, less than 10% of the observations in the test set are stringers, which makes this an unbalanced data set. Fig. 6 shows the resulting confusion matrices for both approaches, dividing the observations into low density and high density observations as previously. We see that the sliding window is very accurate in prediction of low density observations, with an accuracy rate of 0.97. However, only an accuracy of 0.55 is achieved for high density observations. Using the prioritized n-bin, the accuracy on low density observations is slightly decreased, to 0.92, while detection of high density observations is greatly improved, scoring an accuracy of 0.8. The balanced accuracies for the sliding window and the prioritized n-bin are 0.76 and 0.86, respectively. Due to drillstring compression and elongation, measurement error on depth can occur. To account for this, a 1 m acceptance was implemented, meaning that if a prediction is within 1 m of the correct label, it is accepted as correct. The confusion matrices using the 1 m acceptance are shown in Fig. 7. We observe that the results for both approaches are improved. Here, the balanced accuracies are 0.89 for the sliding window and 0.95 for our approach.

## Conclusions

A divided and prioritized experience replay suited for streaming regression has been presented, making use of known ideas such as retention of relevant observations along with prioritization. This makes the model less biased to the distribution of the latest available observations, and results in more frequent sampling of observations where the model performs poorly.

Comparison to a standard sliding window has been made on real-world data. From our results, we can deduce that the standard sliding window results in forgetting of old, rare events, leading to failure to detect them. Especially in cases where exclusively common events have been observed, the model becomes biased towards these observations, failing to detect new, important events. The presented n-bin method, on the other hand, retains observations from the entire range of the prediction space, resulting in more accurate estimates for these observations at a small cost in accuracy on the common events. Also, some false detections are observed. In addition to analyses on the regression formulation, a simplified rephrasing to a binary classification problem has been performed. These results divide the observations into two classes to provide added insight into the improved performance on the rare events.

## Declaration of Competing Interest

The authors declare that they have no known competing financial interests or personal relationships that could have appeared to influence the work reported in this paper.
